# Utility of Patch Testing, LTT, and HLA Genotyping in Identifying Culprit Drugs and Diagnosing Multiple Drug Hypersensitivity in Definite DRESS: A 10‐Year Cohort Study

**DOI:** 10.1002/clt2.70172

**Published:** 2026-04-21

**Authors:** Derya Ünal, Osman Ozan Yeğit, Semra Demir, Bircan Erden, Pelin Korkmaz, Fatma Savran Oğuz, Çiğdem Kekik Çınar, Sonay Temurhan, Deniz Eyice Karabacak, Ilkim Deniz Toprak, Zeynep Kılınç, Mehmet Sait Yordam, Nevzat Kahveci, Merve Hörmet İğde, Şule Çelik Kamacı, Ayşe Feyza Aslan, Mehmet Emin Sezgin, Esra Kaya, Leyla Bölek, Raif Coşkun, Müge Olgaç, Nida Öztop, Aslı Gelincik

**Affiliations:** ^1^ Division of Allergy and Clinical Immunology Department of Internal Medicine Istanbul Faculty of Medicine Istanbul University Istanbul Turkey; ^2^ Department of Medical Biology Istanbul Faculty of Medicine Istanbul University Istanbul Turkey

**Keywords:** Drug Reaction with Eosinophilia and Systemic Symptoms (DRESS) syndrome, human leukocyte antigen (HLA) genotyping, long‐term sequelae, lymphocyte transformation tests, multiple drug hypersensitivity syndrome, patch testing

## Abstract

**Background/Aim:**

We evaluated the role of lymphocyte transformation test (LTT), patch testing (PT), and human leukocyte antigen (HLA) genotyping in identifying culprit drugs in Drug Reaction with Eosinophilia and Systemic Symptoms (DRESS) and assessed long‐term outcomes.

**Methods:**

DRESS was diagnosed using Registry of Severe Cutaneous Adverse Reactions (RegiSCAR) criteria; only definite cases (score > 5) were included. Severe DRESS was defined as ≥ 1 major organ involvement with clinical and laboratory abnormalities, and RegiSCAR scores supported severity assessment. Patients were grouped by single‐ or multiple drug exposure. Drug causality was assessed using the WHO‐UMC criteria. LTT, PT, and/or HLA genotyping were performed for “possible” culprit drugs. Multiple drug hypersensitivity syndrome (MDHS) was defined as hypersensitivity to ≥ 2 unrelated drugs confirmed by testing. Hypersensitivity to a single drug was classified as single‐drug hypersensitivity (SDH). Long‐term follow‐up assessed autoimmune diseases and neo‐sensitization to unrelated drugs.

**Results:**

Fifty patients with definite DRESS (median age 49 years; 50% were female) were included, and six (12%) had severe disease. Compared with non‐severe cases (mild/moderate), severe cases had higher median RegiSCAR scores (8 [7–8] vs. 6 [6–7]; *p* = 0.012) and more frequent facial edema (*p* < 0.001) and lymphadenopathy (*p* = 0.029). MDHS was more common in severe cases (83% vs. 11%; *p* = 0.001), and HLA risk alleles were more prevalent (*p* = 0.016), including HLA‐B*58:01, HLA‐A*31:01, and HLA‐B*13:02, associated with allopurinol, carbamazepine, and levofloxacin‐induced DRESS, respectively. Among 20 patients with multiple drug exposures, 10 were diagnosed with MDHS: 5 simultaneous, 2 sequential, and 4 long‐interval; one patient exhibited both simultaneous and long‐interval reactions. Compared with SDH, MDHS patients had higher median RegiSCAR scores (7 [6–8] vs. 6 [6–8]; *p* < 0.001) and a higher prevalence of HLA risk alleles (*p* = 0.038), including HLA‐B*58:01 (allopurinol), HLA‐A*31:01 (carbamazepine), HLA‐B*13:02 (levofloxacin) and HLA‐A*24:02 (lamotrigine). Long‐term complications occurred in eight patients: four developed autoimmune diseases, and four had neo‐sensitization leading to subsequent DRESS, considered long‐interval MDHS.

**Conclusion:**

PT and LTT effectively identified culprit drugs in DRESS, particularly in multiple drug exposure. MDHS was more frequent in severe DRESS and associated with a higher prevalence of drug‐related HLA risk alleles; however, no single HLA marker defined MDHS. Long‐term follow‐up was essential to detect autoimmune sequelae and neo‐sensitization, and to guide safe drug evaluation and reintroduction.

## Graphical abstract

In a prospective cohort of patients with definite Drug Reaction with Eosinophilia and Systemic Symptoms (DRESS), multiple drug hypersensitivity syndrome (MDHS) was associated with increased disease severity and a higher frequency of human leukocyte antigen (HLA) risk alleles. Patch testing and lymphocyte transformation test (LTT) helped identify culprit drugs. Long‐term follow‐up revealed autoimmune sequelae and neo‐drug sensitization. DRESS, Drug Reaction with Eosinophilia and Systemic Symptoms; HLA, Human leukocyte antigen; LTT, Lymphocyte transformation test; MDHS, Multiple drug hypersensitivity syndrome.

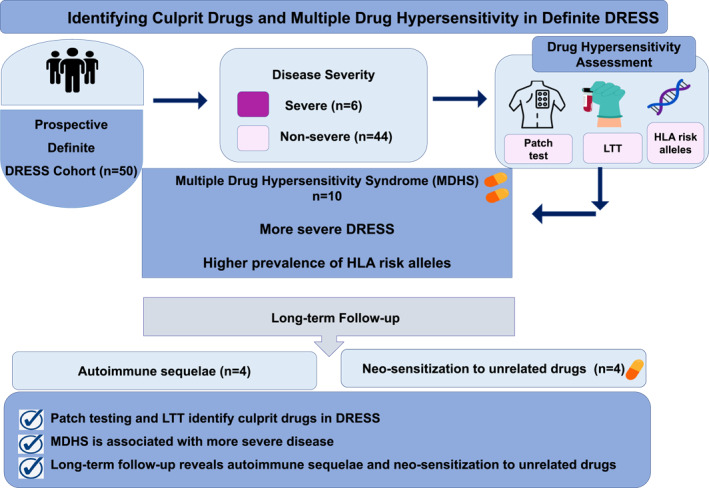

**Abbreviations**
CI: confidence interval
CMV: cytomegalovirus
CSU: chronic spontaneous urticaria
DRESS: Drug Reaction with Eosinophilia and Systemic Symptoms
EBV: epstein‐barr virus
HHV: human herpesvirus
HLA: human leukocyte antigen
IL‐5: interleukin 5
IL‐5R: interleukin 5 receptor
LTT: lymphocyte transformation test
MDHS: multiple drug hypersensitivity syndrome (study‐specific)
OR: odds ratio
PT: patch test
RegiSCAR: registry of severe cutaneous adverse reactions
SDH: single drug hypersensitivity syndrome (study‐specific)
WHO‐UMC: World Health Organization–Uppsala Monitoring Center

## 1 Introduction

Drug Reaction with Eosinophilia and Systemic Symptoms (DRESS) is a rare but potentially life‐threatening severe cutaneous adverse drug reaction (SCAR) characterized by fever, skin rash, lymphadenopathy, hematologic abnormalities, and visceral organ involvement [1]. The clinical spectrum ranges from mild symptoms to severe cases associated with increased morbidity and mortality. Prolonged latency and clinical variability often result in delayed diagnosis, thereby increasing the risk of relapse upon re‐exposure. To ensure accurate diagnosis, structured tools such as the Registry of Severe Cutaneous Adverse Reactions (RegiSCAR) system are widely used, applying a two‐step framework with separate inclusion and validation (scoring) criteria. Diagnostic classification is based on the validation score, and importantly, rash is not mandatory; different combinations of clinical and laboratory features are allowed, reflecting the heterogeneity of DRESS [2]. The RegiSCAR score facilitates standardized diagnosis, supports the assessment of disease severity, and informs clinical decision‐making [3]. Notably, RegiSCAR provides a structured framework for diagnosis, although it does not by itself definitively establish drug causality [2, 3].

Early recognition and prompt withdrawal of the culprit drug are therefore essential. However, identifying the culprit drug remains particularly challenging, especially in patients exposed to multiple drugs concurrently [1].

In vitro tests, such as the lymphocyte transformation test (LTT) and the enzyme‐linked immunospot assay (ELISpot), are often recommended before in vivo tests, as in vivo tests carry a risk of systemic hypersensitivity reactions that may lead to disease flare or relapse. However, the diagnostic sensitivity and specificity of these in vitro tests remain incompletely validated [4, 5, 6]. Furthermore, they are primarily employed in research settings and are not routinely available in all clinical centers.

When in vitro tests are negative or unavailable, patch testing (PT) carries a relatively low risk of systemic reactions and may be considered after complete resolution of the acute phase in selected cases, especially in multidrug‐exposed patients. It may provide supportive information for identifying the culprit drug or assessing potential cross‐reactivity [6]. However, the diagnostic sensitivity of PT in DRESS is variable and generally limited, with reported positivity rates of approximately 50% at best in some studies. Because of the limited sensitivity of PT in DRESS, drug provocation testing (DPT) cannot be recommended solely based on a negative test result [7, 8].

According to current guidelines and recommendations, in vivo testing is generally contraindicated in SCAR. The available evidence mainly originates from specialized research centers. In this context, intradermal testing (IDT) may be considered only in highly selected cases in specialized drug allergy centers under careful supervision, whereas DPT is contraindicated because of the potential for relapse [6, 9, 10, 11, 12].

Human leukocyte antigen (HLA) genotyping has been associated with certain drug‐induced severe cutaneous adverse reactions; however, its clinical utility in patients with DRESS remains incompletely defined. Carriage of a specific HLA risk allele does not necessarily result in disease, and the predictive value of HLA testing varies depending on the drug and the population studied. Moreover, the number needed to test differs considerably across HLA–drug associations. Important limitations of HLA testing include incomplete penetrance, variable positive predictive value, and significant population and drug‐specific heterogeneity. Therefore, HLA genotyping should be interpreted as a supportive rather than definitive tool in the assessment of genetic susceptibility [9, 10, 11].

Multiple drug hypersensitivity syndrome (MDHS) is relatively common in DRESS cases, particularly within SCAR associated with multiple drug exposure. It is defined as hypersensitivity reactions to two or more unrelated drugs and is classified into simultaneous, sequential, or long interval patterns [4, 5, 6, 11]. MDHS occurs more frequently in severe DRESS, with risk factors including drug type, high cumulative dose, and prolonged use [11]. HLA genotyping may provide supportive information in patients exposed to multiple potential culprit drugs, particularly when a drug with a well‐established HLA association is involved, helping to identify the most likely responsible drug. In patients with confirmed MDHS, the initial reaction may also be associated with a known drug‐specific HLA risk allele. However, MDHS itself has not been linked to a common or defining HLA marker [7].

Long‐term sequelae of DRESS include the development of autoimmune diseases and neo‐sensitization leading to subsequent DRESS, emphasizing the importance of prolonged patient follow‐up [13].

We aimed to evaluate the role of LTT, PT, and HLA genotyping in patients with DRESS, particularly those with multiple drug exposures. In addition, we described the characteristics of MDHS in our local cohort and documented long‐term sequelae.

## 2 Methods

### 2.1 Study Design and Population

This observational study was conducted at the Adult Allergy Clinic of Istanbul Faculty of Medicine between 2010 and 2024, with patients prospectively followed; ethics approval (Istanbul University, 2025:93) and informed consent were obtained from participants. Among 90 patients with drug‐induced eruptions, 50 who met the RegiSCAR criteria for definite DRESS (score > 5) and had documented drug exposure within the established 2–8 weeks latency period prior to symptom onset were included.

Demographics, risk factors, drug histories, and reaction resolution dates were recorded. Viral reactivations of Epstein‐Barr virus (EBV), cytomegalovirus (CMV), and human herpesvirus (HHV)‐6 were assessed via quantitative PCR; HHV‐7 testing was unavailable. Skin involvement was assessed as a percentage of body surface area, and biopsies were performed in some patients to confirm the diagnosis and evaluate histopathology.

Patients without documented drug exposure during the established 2–8 weeks latency period prior to symptom onset were excluded.

All patients underwent a comprehensive evaluation to rule out infectious mimickers, including screening for acute viral and relevant bacterial infections. Cases in which clinical and microbiological findings sufficiently explained the presentation as an acute infection were excluded.

Some patients had positive PCR results for EBV or CMV. However, their clinical and laboratory findings were not consistent with an acute primary infection. Therefore, increases in viral load observed after the onset of DRESS were interpreted as secondary viral reactivation associated with DRESS‐related immune dysregulation rather than primary infectious triggers.

Patients who met the diagnostic criteria for DRESS but had no identifiable drug exposure and no evidence of active infection after comprehensive evaluation were classified as idiopathic and excluded [9, 10, 12].

### 2.2 Acute Management of DRESS

Patients presenting with moderate to severe DRESS were treated with systemic corticosteroids, with doses individualized based on disease severity and gradually tapered. Corticosteroids were not administered in patients with active tuberculosis or uncontrolled diabetes mellitus. In these patients, as well as in those with an insufficient clinical response to corticosteroids, alternative treatments were administered, including cyclosporine, intravenous immunoglobulin (IVIG), anti‐IL‐5 agent (mepolizumab), anti‐IL‐5 receptor antagonist (benralizumab), and plasmapheresis [9, 14].

### 2.3 Assessment of Disease Severity

Disease severity was classified based on organ involvement and laboratory findings, as adapted from previous studies [9, 12, 15]. Mild cases had minimal or no visceral involvement; moderate cases had at least one organ with moderate dysfunction (e.g., ALT 4–15 × ULN, creatinine > 26.4 μmol/L or 1.5 × baseline, neutrophils 500–1500/μL, platelets 50,000–100,000/μL); severe cases had major organ failure or extreme laboratory abnormalities (e.g., ALT > 15 × ULN, progressive renal failure, hemoglobin < 7 g/dL, neutrophils < 500/μL, hemophagocytosis, or lung, heart, or nervous system involvement). Additionally, RegiSCAR scores were determined for all patients and used as a supplementary tool to support the clinical assessment of disease severity [3, 9, 10]. For comparative analyses, mild and moderate cases were combined into a non‐severe group and compared with severe DRESS.

### 2.4 Identification of Culprit Drugs and Diagnostic Testing

#### 2.4.1 Culprit Drug Identification

Detailed drug histories included all medications and herbal products taken 2–8 weeks prior to symptom onset [12]. Patients were categorized based on single‐ or multidrug exposure. Suspected drugs were evaluated using the WHO‐Uppsala Monitoring Center (WHO‐UMC) causality classification; drugs labeled “probable” underwent further evaluation [16]. MDHS was confirmed by positive LTT/PT in the context of multi‐drug use, and genetic risk was further assessed using HLA allele typing [10, 17, 18].

#### 2.4.2 Diagnostic Tests and Risk Assessment

##### 2.4.2.1 Lymphocyte Transformation Tests

Peripheral blood mononuclear cells were isolated from heparinized blood and cultured according to standard laboratory procedures. Cells were stimulated with a range of concentrations of the suspected drug to assess lymphocyte proliferation. Unstimulated wells served as negative controls, and pokeweed mitogen (PWM, 5 μg/mL) was used as a positive control. Cultures were incubated for 6 days, and lymphocyte proliferation was evaluated by measuring [^3H]‐thymidine incorporation. The stimulation index (SI), defined as the ratio of proliferation in the presence versus the absence of the drug, was considered positive if SI > 3 [17]. This test was performed for diagnostic purposes in a clinical setting, following the laboratory's standard operating procedures.

LTT was performed after complete clinical stabilization, approximately 4–8 weeks after the onset of the acute reaction [17]. Testing was preferentially conducted in patients with severe DRESS, particularly in those with major organ involvement or when precise identification of the culprit drug was clinically essential. The test was not performed in patients with immunosuppressive conditions (including active malignancy or lymphoma), in those receiving ongoing systemic corticosteroid therapy that could not be discontinued, or when patients declined further testing [9, 10, 17]. Due to resource and cost considerations, LTT was selectively applied rather than systematically performed in all patients.

##### 2.4.2.2 Patch Testing

For patients with negative or unavailable LTT, PT was performed at least 6 months after symptom resolution, excluding patients with severe DRESS and those who declined testing.

For antituberculosis drugs, testing was performed earlier due to urgent treatment needs. Commercial tablets were crushed, diluted in petrolatum (1%, 10%, 30%; based on reaction severity and literature recommendations), applied to the upper back, and read at 48 h, 72 h, 96 h, and day 7 [5, 18, 19, 20, 21, 22, 23].

##### 2.4.2.3 HLA Typing

Genomic DNA was extracted from peripheral blood samples using the EZ1 DNA Blood 200 μL Kit (Qiagen, Germany). DNA purity and concentration were determined by spectrophotometry. HLA Class I (HLA‐A, ‐B, ‐C) and Class II (HLA‐DRB1, ‐DQB1, ‐DQA1, ‐DPB1, ‐DPA1) loci were genotyped using the Luminex xMAP PCR‐SSO assay (One Lambda SSO Typing Kit). PCR products were hybridized to fluorescent microspheres for allele detection. All assays included positive and negative controls, and PCR products were verified by gel electrophoresis before hybridization [24].

HLA genotyping was performed according to clinical indications and test availability. As HLA status is genetically determined and remains unaffected by disease activity, genotyping was conducted independently of the timing of the acute episode [21].

### 2.5 Diagnosis of Multiple Drug Hypersensitivity Syndrome

Multiple drug hypersensitivity syndrome was considered in patients with a compatible clinical history, including documented exposure to multiple unrelated drugs, a clear temporal relationship between each drug exposure and symptom onset, characteristic clinical features of drug hypersensitivity, and resolution after drug withdrawal. The diagnosis was supported by positive PT or LTT results to ≥ 2 unrelated agents. MDHS was categorized as:

- **Simultaneous type:** reactions to multiple drugs occurring concurrently or during the same treatment course, with confirmed test reactivity to each drug;
- **Sequential type:** reactions to different drugs occurring on separate occasions, each confirmed by testing;
- **Long‐interval type:** new reactions to unrelated drugs developing months after DRESS resolution, confirmed by testing [11, 23, 25].

Patients with a single positive test result and negative test results for all other drugs were not considered to have MDHS; instead, they were classified as having single‐drug hypersensitivity (SDH).

SDH was diagnosed when DRESS was attributed to one drug, confirmed by positive PT and/or LTT or by a strong clinical history with clear temporal correlation and improvement after discontinuation. Clinical severity and HLA risk alleles were compared between MDHS and SDH groups.

### 2.6 Slow Drug Reintroduction

In selected cases, slow drug reintroduction was considered when the suspected drug was deemed indispensable and no appropriate alternative treatment options were available. This approach was applied only in patients with negative investigations and should not be interpreted as a protocol for confirmed allergic patients.

In patients with tuberculosis or lymphoma, anti‐tuberculosis drugs and chemotherapy agents were reintroduced using slow, stepwise protocols after negative allergy testing. Each anti‐tuberculosis drug was initiated at 1/10,000 of the therapeutic dose and increased at 2–3‐h intervals under inpatient monitoring until the full dose was achieved. For bendamustine chemotherapy, a novel graded reintroduction protocol was developed and implemented.

Drugs were not reintroduced if they were not indispensable, even when negative test results were available [12, 22].

### 2.7 Long‐Term Monitoring

Long‐term outcomes including autoimmunity and neo‐sensitization leading to subsequent DRESS were evaluated in patients followed for at least 1 year. Follow‐up included clinical and laboratory assessments every 6 months (CBC, urinalysis, renal, liver, thyroid function, glucose). Annual assessments included ANA, anti‐TPO, rheumatoid factor, and autologous serum testing. Imaging was performed when clinically indicated; specifically, thyroid ultrasound was performed in a patient with suspected Hashimoto's thyroiditis, and renal ultrasound followed by biopsy confirmed membranous glomerulonephritis in another patient.

During follow‐up, new drug allergies defined as “long‐interval multiple drug hypersensitivity reactions” developing months or years later, were evaluated using the same comprehensive diagnostic approach as the initial DRESS episode. In these patients, DRESS was diagnosed according to the RegiSCAR scoring system (score > 5, indicating definite DRESS). Culprit drugs were confirmed using PT and the LTT, and HLA genotyping was performed to assess drug‐specific genetic susceptibility.

Overall, these follow‐up assessments were performed according to each patient's clinical condition and were in line with previous reports and the EAACI position paper, which recommends long‐term monitoring of autoimmunity, organ function, and potential neo‐sensitization after resolution of the acute phase [9, 10, 13, 26].

## 3 Statistical Analysis

Data were analyzed using SPSS 25.0 (IBM Corp., Armonk, NY, USA). Continuous variables were expressed as median and interquartile range (IQR), and categorical variables as counts and percentages. Comparisons between groups were performed using the Mann–Whitney *U* test for continuous variables and the two‐tailed Fisher's exact test for categorical variables, as appropriate. Univariate logistic regression analysis was conducted to evaluate the association of clinical and laboratory variables with severe DRESS. Odds ratios (ORs) and 95% confidence intervals (CIs) were calculated. *p*‐value < 0.05 was considered statistically significant.

## 4 Results

### 4.1 Demographics and Clinical Characteristics

A total of 90 patients with drug‐induced eruptions were screened, of whom 50 met the RegiSCAR criteria for definite DRESS (score > 5) and were included in the study (median age, 49 years; range, 19–80 years; 25/50 [50%] female). Common comorbidities included 8/50 (16%) with malignancy, 7/50 (14%) with epilepsy, 5/50 (10%) with type 2 diabetes and 4/50 (8%) with chronic renal insufficiency. Four patients (4/50 (8%) had a prior history of drug allergy: 1/50 (2%) had maculopapular exanthem (MPE) due to nonsteroidal anti‐inflammatory drugs and 3/50 (6%) had MPE induced by antibiotics. A family history of drug allergy was reported in 2/50 (4%) patients. Over the 10‐year study period, 2/50 (4%) died from unrelated causes (renal failure and lymphoma).

All patients developed a generalized erythematous maculopapular rash affecting more than 50% of the body surface area, dark red to violaceous in color, accompanied by mild‐to‐moderate skin infiltration. Fever occurred in 49/50 (98%) patients, hematologic abnormalities in 50/50 (100%), and hepatic involvement in 30/50 (60%). Other systemic manifestations and viral reactivations are summarized in Table 1.

**TABLE 1:** Demographic and clinical characteristics of the study patients.

Characteristics	*n* (%)
Age (years), median (range)	49 (19–80)
Gender, female, *n* (%)	25 (50)
Clinical manifestations	
RegiScar score, median (IQR)	6 (5–8)
Severe DRESS, *n* (%)	6 (12)
Presence of DRESS‐associated HLA risk alleles, *n*/*N* (%)	15/37 (40)
Viral reactivation, *n*/*N* (%)	14/50 (28)
EBV	6/50 (12)
CMV	8/50 (16)
HHV‐6	0/6 (0)
Cutaneous involvement	
Body surface area involvement (BSA), % median (IQR)	63 (54–81)
Mucosal involvement, *n* (%)	3 (6)
Facial edema, *n* (%)	10 (20)
Systemic involvement	
Fever, *n* (%)	49 (98)
Lymphadenopathy, *n* (%)	7 (14)
Hematological involvement, *n* (%)	50 (100)
Eosinophilia (> 700 eosinophils/mm^3^)	40 (80)
Lymphopenia (< 1500/μL)	12 (24)
Lymphocytosis (> 4000/μL)	11 (22)
Thrombocytopenia (< 150,000/μL)	10 (20)
Hepatic involvement, *n* (%)	30 (60)
Renal involvement, *n* (%)	2 (4)
Cardiac, pulmonary, pancreatic involvement, *n* (%)	0

Abbreviations: BSA, body surface area; CMV, cytomegalovirus; EBV, Epstein‐Barr virus; HHV‐6, human herpesvirus 6; IQR, interquartile range.

### 4.2 Management of DRESS

Monotherapy was administered to 39 patients, including corticosteroids (*n* = 34), mepolizumab (*n* = 4), and benralizumab (*n* = 1). Combination therapies were used in 11 patients, including corticosteroid + IVIG (*n* = 6), corticosteroid + cyclosporine (*n* = 2), corticosteroid + cyclosporine + IVIG (*n* = 1), corticosteroid + cyclosporine + IVIG + plasmapheresis (*n* = 1), and mepolizumab + IVIG (*n* = 1). CMV reactivation occurred in eight patients and EBV reactivation in six patients. One patient with high CMV viral load was treated with ganciclovir. No HHV‐6 reactivation was detected.

### 4.3 Disease Severity

A total of 50 patients with DRESS were analyzed, including 44 with non‐severe and 6 with severe disease. Compared with non‐severe cases, severe cases had a higher median RegiSCAR score (8 [7–8] vs. 6 [6–7], *p* = 0.012). Facial edema (*p* < 0.001) and lymphadenopathy (*p* = 0.029) were more common in severe cases. MDHS occurred more frequently in severe cases (83%) than in non‐severe cases (11%; *p* = 0.001). The prevalence of DRESS‐associated HLA risk alleles was higher in severe cases (*p* = 0.016). Identified alleles included HLA‐B*58:01, HLA‐A*31:01, and HLA‐B*13:02, previously reported to be associated with allopurinol, carbamazepine, and levofloxacin‐induced DRESS, respectively. No other variables differed significantly between groups. The comparison of characteristics between non‐severe and severe DRESS is shown in Table 2.

**TABLE 2:** Comparison of characteristics between non‐severe (mild and moderate) and severe DRESS.

Characteristics	Non severe DRESS (*n* = 44)	Severe DRESS (*n* = 6)	*p* value
Age years, median (range)	50 (19–80)	34 (20–78)	NS
Gender, male *n* (%)	20 (45)	5 (83)	NS
RegiSCAR score, median (IQR)	6(6–7)	8 (7–8)	***p* = 0.012**
Multiple drug hypersensitivity syndrome, *n* (%)	5 (11)	5 (83)	***p* = 0.001**
Presence of DRESS‐associated HLA risk alleles, *n*/*N* (%)	10/31 (32)	5/6 (83)	***p* = 0.016**
Cutaneous involvement			
Body surface area (BSA) involvement, % median (IQR)	63 (54–81)	68 (61–81)	NS
Mucosal involvement	1 (2)	2 (33)	*p* = 0.324
Facial edema	5 (11)	5 (83)	***p* < 0.001**
Viral reactivation			
EBV	4 (9)	2 (33)	NS
CMV	7 (16)	1 (17)	NS
HHV‐6	0	0	NS
Systemic involvement			
Fever	44 (100)	5 (83)	NS
Lymphadenopathy	4 (9)	3 (50)	***p* = 0.029**
Hematological involvement			
Eosinophilia (> 700 eosinophils/mm^3^)	34 (77)	6 (100)	NS
Lymphopenia (< 1500/μL)	9 (20)	3 (50)	NS
Lymphocytosis (> 4000/μL)	10 (23)	1 (17)	NS
Thrombocytopenia (< 150,000/μL)	9 (20)	3 (50)	NS
Organ involvement			
Hepatic involvement, *n*(%)	25 (56)	5 (83)	NS
Renal involvement, *n*(%)	2 (4.5)	0	NS
Cardiac, pulmonary, pancreatic involvement	0	0	0

*Note:* Bold values indicate statistically significant differences (*p* < 0.05).

Abbreviations: BSA, body surface area; CMV, cytomegalovirus; EBV, Epstein‐Barr virus; HHV‐6, human herpesvirus 6; IQR, interquartile range; NS, not significant.

### 4.4 Culprit Drug Identification

#### 4.4.1 Multiple Drug Exposures

Among 20 patients with multiple drug exposures, 46 PTs and 26 LTTs were performed. Slow drug reintroduction was selectively performed with 26 drugs that had shown negative test results and was well tolerated.

HLA risk alleles were identified in 11 alleles across 10 patients, including HLA‐B*58:01 (allopurinol, *n* = 3), HLA‐A*31:01 (carbamazepine, *n* = 2), HLA‐B*13:02 (levofloxacin, *n* = 2), HLA‐A*24:02 (lamotrigine, *n* = 1), HLA‐Cw*04:01 (antituberculosis drugs, *n* = 1), HLA‐A*32:01 (vancomycin, *n* = 1), and HLA‐A*02:06 (bendamustine, *n* = 1).

Notably, recurrent HLA alleles were observed among patients exposed to the same culprit drugs, antituberculosis agents and amoxicillin–clavulanate, representing novel drug‐HLA associations not previously described in the literature (Table 3).

**TABLE 3:** HLA Genotypes and Associated Culprit Drugs in DRESS patients.

PN	HLA‐A	HLA‐B	HLA‐C	HLA‐DRB1	HLA‐DQB1	HLA‐DQA1	Culprit drug
1	A*03:01 A*30:01	B*13:02 B*51:01			DQB1*02:02 DQB1*03:02	DQA1* 02:01 DQA1* 03:01	Ethambutol
2	A*03:01 A*24:02	B*55:01 B*58:01				DQA1*01:01 DQA1*01:02	Ethambutol, para‐aminosalicylic acid, cycloserine
3	A*24:02 A*29:01	B*18:01 B*58:01	Cw*07:01 Cw*12:03		DQB1*03:01 DQB1*03:02	DQA1*03:01 DQA1*05:05	Isoniazid, ethambutol
4	A*02:01 A*32:01	B*35:03 B*51:01	Cw*12:03 Cw*16:02	DRB1*13:01 DRB1*16:02	DQB1*05:02 DQB1*06:03	DQA1*01:02 DQA1*01:03	Isoniazid, ethambutol
5	A*24:02	B*51:01 B*55:01		DRB1*04:03 DRB1*13:01	DQB1*03:02 DQB1*06:03	DQA1*01:03 DQA1*03:01	Isoniazid
14					DQB1*03:02 DQB1*05:01		Amoxicillin‐clavulanate
16	A*02:01 A*32:01				DQB1*03:01 DQB1*05:01	DQA1*01:01 DQA1*05:05	Isoniazid, ethambutol
20		B*51:01		DRB1*04:02 DRB1*11:01	DQB1*03:02		Amoxicillin‐clavulanate

Based on the results of these tests and the clinical history of DRESS episodes, patients were subsequently classified as having SDH or MDHS.

Among the 20 patients with multiple drug exposure:

- Six patients with positive results for two or more drugs were classified as MDHS.
- Nine patients with single‐drug test positivity included two patients with two DRESS episodes, classified as MDHS, and seven patients with a single DRESS episode, classified as SDH.
- Of the five patients with negative tests, two who had experienced two DRESS episodes were classified as MDHS, and one patient who had self‐administered one of the suspected drugs at home without any adverse reaction was classified as SDH. The remaining two patients with multiple drug exposure remained unclassified.

Diagnostic test results in DRESS cases Induced by Concurrent Use of Multiple Drugs are summarized in Table 4.

**TABLE 4:** Diagnostic test results in DRESS cases induced by concurrent use of multiple drugs.

PN	Suspected culprit drugs	LTT (+)	LTT (−)	PT positive	PT negative	Slow drug reintroduction/tolerated	HLA associated	Dress episode	Hypersensitivity type
1	Isoniazid, ethambutol, rifampicin, pyrazinamide		Isoniazid, ethambutol, rifampicin, pyrazinamide	Ethambutol	Isoniazid, rifampicin, pyrazinamide	Isoniazid, rifampicin, pyrazinamide	Not associated	Two	MDH
	Levofloxacin	NP	NP		NP	NP	HLA‐B*13:02		
2	Ethambutol, para‐aminosalicylic acid, cycloserine, pyrazinamide, moxifloxacin	NP	NP	Ethambutol, para‐aminosalicylic acid, cycloserine	Pyrazinamide, moxifloxacin	Pyrazinamide, moxifloxacin	Not associated	One	MDH
3	Isoniazid, ethambutol, rifampicin, pyrazinamide	NP	NP	Isoniazid, ethambutol	Rifampicin, pyrazinamide	Rifampicin, pyrazinamide		Two	MDH
	Allopurinol						HLA‐B*58:01		
4	Isoniazid, ethambutol, rifampicin, pyrazinamide		Isoniazid, ethambutol, rifampicin, pyrazinamide	Isoniazid, ethambutol	Rifampicin, pyrazinamide	Rifampicin, pyrazinamide	Not associated	One	MDH
5	Isoniazid, ethambutol, rifampicin, pyrazinamide	NP	NP	Isoniazid	Ethambutol, rifampicin, pyrazinamide	Ethambutol, rifampicin, pyrazinamide	Not associated	One	SDH
6	Ethambutol, para‐aminosalicylic acid, cycloserine, pyrazinamide, moxifloxacin	NP	NP	Cycloserine	Ethambutol, para‐aminosalicylic acid, pyrazinamide, moxifloxacin	Ethambutol, para‐aminosalicylic, pyrazinamide, moxifloxacin	Not associated	One	SDH
7	Isoniazid, ethambutol, rifampicin, pyrazinamide	NP	NP	Isoniazid	Ethambutol, rifampicin, pyrazinamide	Ethambutol, rifampicin, pyrazinamide	Not associated	One	SDH
8	Isoniazid, ethambutol, rifampicin, pyrazinamide	NP	NP	Ethambutol	Isoniazid, rifampicin, pyrazinamide	Isoniazid, rifampicin, pyrazinamide	Not associated	One	SDH
9	Isoniazid, ethambutol, rifampicin, pyrazinamide	NP	NP		Isoniazid, ethambutol, rifampicin, pyrazinamide	NP	HLA‐Cw*04:01	One	Unclassified
10	Allopurinol, N‐Acetylcysteine		Allopurinol	NP	NP	Self‐administered N‐acetylcysteine	HLA‐B*58:01	Two	MDH
	Levofloxacin		Levofloxacin	NP	NP	NP	HLA‐B*13:02		
11	Carbamazepine, lamotrigine	Carbamazepine	Lamotrigine		Carbamazepine, lamotrigine	NP	HLA‐A* 31:01	One	SDH
12	Carbamazepine levetiracetam	Carbamazepine, levetiracetam		NP	NP	NP	HLA‐A*31:01	Two	MDH
	Lamotrigine, topiramate	Lamotrigine, topiramate		NP	NP	NP	HLA‐A*24:02		
13	Bendamustine, brentuximab	NP	NP		Bendamustine, brentuximab	Bendamustine	HLA‐A*02:06	One	Unclassified
14	Bendamustine, brentuximab	NP	NP		Bendamustine, brentuximab	Bendamustine	Not associated	Two	MDH
	Amoxicillin‐clavulanate	NP	NP	NP	NP	NP	Not associated		
15	Moxifloxacin, radiocontrast agent	NP	NP		Moxifloxacin	Self‐administered moxifloxacin	Not associated	One	SDH
16	Isoniazid, ethambutol, rifampicin, pyrazinamide	Isoniazid, ethambutol	Rifampicin, pyrazinamide	NP	NP	Rifampicin, pyrazinamide	Not associated	One	MDH
17	Amoxicillin‐clavulanate, metronidazole	Amoxicillin‐clavulanate, metronidazole	NP	NP	NP	NP	Not associated	One	MDH
18	Trimethoprim‐sulfamethoxazole, vancomycin	NP	NP	Vancomycin	Trimethoprim‐sulfamethoxazole	NP	HLA‐A*32:01	One	SDH
19	Trimethoprim‐sulfamethoxazole	Trimethoprim‐sulfamethoxazole	Allopurinol	NP	NP	NP		Two	MDH
	Allopurinol						HLA‐B*58:01		
20	Vitamin C, amoxicillin/clavulanic acid	Vitamin C	Amoxicillin‐clavulanate	NP	NP	NP	Not associated	One	SDH

Abbreviations: HLA, human leukocyte antigen; LTT, lymphocyte transformation test; MDHS, multiple drug hypersensitivity syndrome; NP, not performed; PN, Patient number; PT, patch test; SDH, single‐drug hypersensitivity.

#### 4.4.2 Single‐Drug Exposures

Among 30 patients with single‐drug exposure, 10 PTs and 2 LTTs were performed. HLA typing in 17 patients revealed risk alleles, including HLA‐A*32:01 (vancomycin, *n* = 2) and HLA‐A*31:01 (carbamazepine, *n* = 2). All patients with single‐drug exposures were classified as SDH. The diagnostic test results in DRESS cases induced by single‐drug use are shown in Table 5.

**TABLE 5:** Diagnostic test results in DRESS cases induced by single drug use.

PN	Culprit drug	LTT (+)	LTT (−)	PT positive	PT negative	DPT tolerated	HLA associated
21	Carbamazepine	Carbamazepine			Carbamazepine	NP	HLA‐A* 31:01
22	Sulfasalazine	NP			Sulfasalazine	NP	Not associated
23	Radiocontrast agent	NP			Radiocontrast agent	NP	Not associated
24	Vancomycin	NP		NP		NP	HLA‐A*32:01
25	Methylprednisolone	NP			Methylprednisolone	NP	Not associated
26	Trimethoprim sulfamethoxazole	NP		NP		NP	Not associated
27	Carbamazepine	NP		NP		NP	Not associated
28	Ramipril	NP		NP		NP	Not associated
29	Moxifloxacin	NP		NP		NP	Not associated
30	Sulfasalazine	NP			Sulfasalazine	NP	Not associated
31	Ciprofloxacin	NP		Ciprofloxacin		NP	Not associated
32	Dexketoprofen	NP			Dexketoprofen	NP	Not associated
33	Amoxicillin–clavulanate	NP			Amoxicillin‐clavulanate	NP	Not associated
34	Vancomycin	NP		NP		NP	HLA‐A*32:01
35	Carbamazepine	NP			Carbamazepine	NP	Not associated
36	Carbamazepine	NP			Carbamazepine	NP	HLA‐A* 31:01
37	Vancomycin		Vancomycin	NP		NP	Not associated

Abbreviations: DPT, drug provocation test; HLA, human leukocyte antigen; LTT, lymphocyte transformation test; NP, not performed; PN, Patient number.

### 4.5 Multiple Drug Hypersensitivity Syndrome

Ten patients were diagnosed with MDHS. Five patients had simultaneous reactions, two had sequential reactions, and four had long‐interval reactions. One patient experienced both simultaneous and long‐interval MDHS, resulting in a total of 11 MDHS reactions. The clinical features of patients with MDHS are shown in Table 6.

**TABLE 6:** Clinical features of MDHS patients.

PN	MDHS	Episode	Culprit drug	Interval	Positive/negative (PT and LTT)	HLA
2	Simultane	1st	Ethambutol, para‐aminosalicylic acid, cycloserine	Same time	PT: Ethambutol (+), para‐aminosalicylic acid (+), cycloserine (+) pyrazinamide (−), moxifloxacin (−)	Not associated
4	Simultane	1st	Isoniazid, ethambutol	Same time	PT: Isoniazid (+), ethambutol (+) rifampicin (−), pyrazinamide (−)	Not associated
16	Simultane	1st	Isoniazid, ethambutol	Same time	LTT: Isoniazid (+), ethambutol (+) rifampicin (−), pyrazinamide (−)	Not associated
12	Simultane	1st	Carbamazepine, levetiracetam	Same time	LTT: Carbamazepine (+), levetiracetam (+)	HLA‐A*31:01
	Simultane + long‐interval	2nd	Lamotrigine topiramate	Same time	LTT: Lamotrigine (+), topiramate (+)	HLA‐A*24:02
17	Simultane	1st	Amoxicillin–clavulanate, metronidazole	Same time	LTT: Amoxicillin–clavulanate (+), metronidazole (+)	Not associated
3	Sequential	1st	Isoniazid, ethambutol	1 month	PT: Isoniazid (+), ethambutol (+) rifampicin (−), pyrazinamide (−)	
		2nd	Allopurinol		Allopurinol: NP	HLA‐B*58:01
19	Sequential	1st	TMP‐SMX	1 month	LTT: TMP‐SMX (+)	
		2nd	Allopurinol		Allopurinol (−)	HLA‐B*58:01
1	Long‐interval	1st	Ethambutol	1 year	PT: Ethambutol (+), isoniazid (−), rifampicin (−), pyrazinamide (−)	
		2nd	Levofloxacin		Levofloxacin: NP	HLA‐B*13:02
10	Long‐interval	1st	Allopurinol	3 years	LTT: Allopurinol (−)	HLA‐B*58:01
		2nd	Levofloxacin		Levofloxacin (−)	HLA‐B*13:02
13	Long‐interval	1st	Amoxicillin–clavulanate	7 years	Amoxicillin–clavulanate: NP	Not associated
		2nd	Bendamustine brentuximab		PT: Bendamustine (−) Brentuximab (−)	

*Note:* Patient 12 experienced simultaneous MDH with carbamazepine and levetiracetam and three years later had another simultaneous MDH with and lamotrigine and topiramate; therefore, this patient was classified into both the simultaneous and long‐term MDH groups.

Abbreviations: HLA, human leukocyte antigen; IDT, Intradermal test; LTT, lymphocyte transformation test; MDHS, multiple drug hypersensitivity syndrome; NP, not performed; PN, Patient number.

#### 4.5.1 Simultaneous MDHS

Five patients developed simultaneous MDHS. In patient 2, PT was positive for ethambutol, para‐aminosalicylic acid, and cycloserine. In patient 4, PT was positive for isoniazid and ethambutol whereas, in patient 16, LTT was positive for same drugs. In these patients, anti‐tuberculosis drugs with negative test results were successfully reintroduced using slow drug reintroduction.

Patient 12 presented with simultaneous MDHS with positive LTT results for carbamazepine and levetiracetam. Notably, 3 years later, she experienced another episode of simultaneous MDHS with lamotrigine and topiramate, representing a long‐interval MDHS pattern. She also carried the risk alleles HLA‐A*31:01 and HLA‐A*24:02, associated with carbamazepine‐ and lamotrigine‐induced hypersensitivity. In patient 17, LTT was positive for amoxicillin–clavulanate and metronidazole.

#### 4.5.2 Sequential MDHS

Two patients developed sequential reactions. PT confirmed isoniazid and ethambutol as culprit drugs in the first episode of one patient, whereas in the other patient LTT identified trimethoprim‐sulfamethoxazole as the culprit drug in the first episode. In both cases, allopurinol triggered the second episode. Identification of the culprit drug was based on the temporal relationship between drug exposure and symptom onset and clinical improvement after drug discontinuation, with the presence of HLA‐B*58:01 providing additional supportive evidence.

#### 4.5.3 Long‐Interval MDHS

Four patients developed long‐interval MDHS to structurally unrelated drugs weeks to years after the initial DRESS episode. One of these patients (patient 12) is described above in the simultaneous MDHS section. In patient 1, PT confirmed ethambutol as the culprit in the first episode; PT with other anti‐tuberculosis drugs was negative, and slow drug reintroduction was successfully performed. Levofloxacin was implicated in the second episode based on the temporal relationship between drug exposure and symptom onset and clinical improvement after drug discontinuation. This patient carried HLA‐B*13:02, previously associated with levofloxacin hypersensitivity.

In patient 10, reactions occurred after exposure to allopurinol and subsequently levofloxacin; LTT results were negative for both drugs. Allopurinol and levofloxacin were implicated in the first and second episodes, respectively, based on the temporal relationship between drug exposure and symptom onset and clinical improvement after drug discontinuation. The patient carried HLA‐B*58:01 and HLA‐B*13:02, associated with allopurinol and levofloxacin hypersensitivity.

In patient 13, reactions occurred after exposure to amoxicillin‐clavulanate and later bendamustine and brentuximab; diagnostic tests were negative or not performed, and the culprit drugs were identified based on the temporal relationship between drug exposure and symptom onset and clinical improvement after drug discontinuation.

### 4.6 Comparison Between MDHS and SDH

Among 50 patients, 38 had SDH and 10 had MDHS, while two patients remained unclassified. Compared with SDH, MDHS patients had a significantly higher median RegiSCAR score (7 [6–8]) than SDH patients (6 [6–8]) (*p* < 0.001). Facial edema (*p* < 0.001) and lymphadenopathy (*p* = 0.042) were more common in MDHS. MDHS was also associated with a higher frequency of relevant HLA risk alleles (*p* = 0.038), including HLA‐B*58:01 (allopurinol), HLA‐A*31:01 (carbamazepine), HLA‐B*13:02 (levofloxacin) and HLA‐A*24:02 (lamotrigine), all previously reported to be associated with drug‐specific DRESS.

Univariate logistic regression analysis showed strong associations of MDHS with severe DRESS (OR = 37.0, 95% CI 4.0–343), facial edema (OR = 27.2, 95% CI 5.5–135), lymphadenopathy (OR = 7.8, 95% CI 1.3–45), and presence of relevant HLA alleles (OR = 7.4, 95% CI 1.4–39). No significant associations were found for age, gender, viral reactivation (EBV, CMV, HHV‐6), mucosal involvement, eosinophilia, lymphopenia, lymphocytosis, thrombocytopenia, or internal organ involvement (hepatic, renal, cardiac, pulmonary, pancreatic).

The differences between MDHS and SDH patients are shown in Table 7.

**TABLE 7:** Comparison of patient characteristics between multiple drug hypersensitivity syndrome and single drug hypersensitivity groups.

Characteristics	SDH (*n* = 38)	MDH (*n* = 10)	*p* value
Demographics			
Age years, median (range)	50 (19–80)	40.50 (20–78)	NS
Gender, female, *n*(%)	21 (55)	4 (40)	NS
Clinical manifestations			
RegiSCAR score, median (IQR)	6 (6–8)	7 (6–8)	***p* < 0.001**
Severe DRESS, *n*(%)	1 (3)	5 (50)	***p* < 0.001**
Presence HLA of allele associated with drug induced‐DRESS, *n*/*N* (%)	6/25 (24)	7/10 (70)	***p* = 0.038**
Viral reactivation, *n* (%)			
EBV	5 (13)	1 (10)	NS
CMV	6 (15)	2 (20)	NS
HHV‐6	0	0	NS
Cutaneous involvement			
% BSA, body surface area involvement, median (IQR)	63 (54–81)	67.5 (54–81)	NS
Mucosal involvement	1 (3)	2 (20)	NS
Facial edema	3 (8)	7 (70)	**p < 0.001**
Systemic involvement			
Fever, *n* (%)	37 (97)	10 (100)	NS
Lymphadenopathy	3 (8)	4 (40)	***p* = 0.042**
Eosinophilia (> 700 eosinophils/mm^3^)	30 (79)	10 (100)	NS
Lymphopenia (< 1500/μL)	7 (18)	5 (50)	NS
Lymphocytosis (> 4000/μL)	10 (26)	1 (10)	NS
Thrombocytopenia (< 150,000/μL)	7 (18)	3 (30)	NS
Hepatic involvement, *n* (%)	24 (63)	6 (60)	NS
Renal involvement	2 (5)	0	NS
Cardiac, pulmonary, pancreatic involvement	0	0	0

*Note:* Bold values indicate statistically significant differences (*p* < 0.05).

Abbreviations: BSA, body surface area; CMV, cytomegalovirus; DRESS, Drug Reaction with Eosinophilia and Systemic Symptoms; EBV, Epstein‐Barr virus; HHV‐6, human herpesvirus 6; HLA, human leukocyte antigen; IQR, interquartile range; MDH, multiple drug hypersensitivity; NS, not significant; SDH, single drug hypersensitivity.

### 4.7 Long‐Term Outcomes

During a 10‐year follow‐up, four patients developed autoimmune sequelae including autoimmune thyroiditis (*n* = 2, within 1 year), chronic spontaneous urticaria (CSU) (*n* = 1, within 2 years), and membranous glomerulonephritis (*n* = 1, within 3 years). None of these patients had severe DRESS during the acute episode, and all were free of autoimmune disease prior to the initial DRESS reaction.

In the same follow‐up period, long‐interval MDHS was observed in four patients, occurring months to years after the initial DRESS episode.

Two of these patients had experienced severe DRESS during the acute phase, while the other two had non‐severe. All four carried at least one drug‐associated HLA allele corresponding to the implicated medications, and one patient carried two different alleles linked to separate culprit drugs.

## 5 Discussion

In this study of 50 DRESS patients prospectively followed, we provide a comprehensive analysis of clinical features, culprit drugs, diagnostic testing, HLA associations, and long‐term outcomes, particularly in the context of multidrug exposure. Consistent with previous studies, most patients were aged 40–50 years. Facial edema was observed in 83% of severe cases, underscoring its role as a defining feature of disease severity [27]. Lymphadenopathy, including mediastinal involvement in two patients, further highlighted the extent of systemic involvement in severe reactions [28]. Mucosal involvement was observed in 6% of cases [29]. Eosinophil counts did not correlate with disease severity in our cohort, in contrast to the findings reported by Pozzo‐Magaña et al. [30].

Kardaun et al. [2] described hepatic involvement, ranging from mild to severe, as a major determinant of DRESS severity. In our cohort, hepatic involvement was similarly frequent and occurred across a comparable spectrum. Notably, in one patient, transaminase levels increased 15‐fold, highlighting the importance of close laboratory monitoring and prompt drug withdrawal [31]. Renal involvement was less common and primarily associated with allopurinol, reflecting its known nephrotoxic risk in DRESS [32]. EBV and CMV reactivation were observed, whereas HHV‐6 was not detected. EBV reactivation caused atypical lymphocytosis and lymphadenopathy in one patient, while CMV reactivation exacerbated hepatitis in another, consistent with previous report [33].

Overall, hepatic and renal involvement and lymphadenopathy reflected severe systemic reactions and corresponded with the organ‐based parameters outlined in RegiSCAR, highlighting both the clinical significance of these findings and the utility of RegiSCAR as a structured tool for assessing disease severity [3].

Treatment of DRESS in our cohort largely followed current guideline recommendations, including systemic corticosteroids, cyclosporine, IVIG, plasmapheresis, and biologics such as mepolizumab or benralizumab. Notably, IL‐5/IL‐5R blockade appeared particularly beneficial in patients who were steroid‐refractory or had contraindications to corticosteroids, such as those with active tuberculosis or uncontrolled hyperglycemia, consistent with emerging reports supporting targeted therapy in severe or complicated cases [14]. These findings reinforce the notion that individualized treatment strategies are critical in optimizing outcomes and minimizing complications.

Antibiotics, particularly anti‐tuberculosis drugs, antiepileptics and allopurinol were the most frequent triggers of DRESS in this study. Anti‐tuberculosis agents accounted for 20% of DRESS cases, substantially higher than the 1.2% previously reported, highlighting the need for close monitoring during combination therapy [31]. Identifying the culprit drug remains difficult without definitive testing, especially in patients exposed to multiple drugs. A detailed clinical history and careful assessment of the latency period are therefore crucial. In the study, latency periods were carefully evaluated, and antibiotics were found to have a shorter median latency compared with other drug classes. This observation is consistent with a previous report and may reflect the more rapid onset of DRESS in response to certain antibiotic exposures [34].

Santiago et al. demonstrated that PT was safe and useful in confirming the culprit drug in antiepileptic‐induced DRESS, whereas its sensitivity appears lower for other drug classes such as allopurinol and some antibiotics [7]. In our study, PT results were positive for some antituberculosis drugs and antibiotics, whereas negative results were observed for several other agents, such as certain antibiotics, antituberculosis drugs, antiepileptics, and bendamustine [19, 35]. These findings suggest that PT may be a useful tool for identifying culprit drugs in selected cases, although its sensitivity remains limited [7, 10].

LTT results varied by patient and drug. Allopurinol yielded a negative result, whereas isoniazid, rifampicin, and carbamazepine were variably positive. Positive LTT results supported identification of the causative drug, while negative results did not exclude hypersensitivity, reflecting high specificity but limited sensitivity, with an estimated sensitivity of approximately 50% reported in previous studies [4, 8, 18, 36, 37, 38, 39].

In the study, HLA genotyping confirmed several known risk alleles: HLA‐A*31:01 with carbamazepine‐induced DRESS, HLA‐A*24:02 with lamotrigine‐induced DRESS, and HLA‐B*13:02 with levofloxacin‐induced DRESS, consistent with previous studies [40, 41, 42]. HLA‐B*58:01 was present in allopurinol‐induced cases, reaffirming its established link, particularly among patients with renal impairment [43]. Rare associations were also observed, including HLA‐A*02:06 with bendamustine and HLA‐Cw alleles with anti‐tuberculosis‐induced DRESS, expanding the spectrum of potential genetic susceptibility [44, 45].

In addition to well‐established drug‐related risk factors, certain HLA alleles were identified in more than one patient for drugs such as antituberculosis agents and amoxicillin–clavulanate, suggesting potential novel drug‐HLA associations that require confirmation in larger studies. However, the clinical relevance of these findings remains uncertain without comparison to allele frequencies in the general population. Therefore, these observations should be interpreted as preliminary evidence of genetic susceptibility rather than definitive risk markers. Future studies incorporating larger cohorts and population‐based HLA data are warranted to confirm these associations and guide personalized management strategies.

Slow drug reintroduction protocols were performed in selected patients requiring essential tuberculosis treatment with negative allergy tests [9, 12, 22]. Notably, two lymphoma patients developed DRESS following brentuximab and bendamustine; PTs were negative, and bendamustine was successfully reintroduced via slow drug reintroduction protocol.

This study highlights the importance of distinguishing SDH from MDHS. LTT and PT have been used to identify MDHS in several studies. Barbaud et al. reported PT positivity in 64% of DRESS cases, with multidrug reactivity persisting for years in 18% of patients [8]. Pichler et al. identified MDHS in 31 patients, 12 (≈39%) fulfilling DRESS criteria, mainly using LTT with occasional PT [11].

In our cohort, MDHS occurred in 10 of 50 DRESS patients (20%). Patients exhibited different patterns of MDHS, including simultaneous, sequential, and long‐interval forms, highlighting the heterogeneous nature of drug hypersensitivity.

Clinically, MDHS was more frequently observed in patients with severe DRESS and was influenced by drug type, high dose, and prolonged exposure [11]. In our cohort, it occurred predominantly in patients with severe disease and was frequently accompanied by facial edema and lymphadenopathy. It was also more commonly observed among patients receiving combination therapies, particularly antituberculosis and antiepileptic drugs [11, 46]. Notably, in one patient with a history of MDHS triggered by multiple drugs, bendamustine was identified as a new trigger for MDHS‐associated DRESS, which to our knowledge has not been previously reported. These findings highlight the persistence and heterogeneity of MDHS and emphasize the importance of comprehensive testing and cautious drug re‐exposure.

LTT and PT contributed to the identification of MDHS in our cohort; however, as not all patients underwent both tests, no direct comparison between the methods was possible. All affected patients carried HLA alleles linked to at least one culprit drug. Two patients had risk alleles associated with different drugs across two distinct DRESS episodes: one with HLA‐B*58:01 and HLA‐B*13:02 (allopurinol and levofloxacin) and another with HLA‐A*31:01 and HLA‐A*24:02 (carbamazepine and lamotrigine).

Beyond the acute phase, DRESS is increasingly recognized as a trigger for long‐term immune dysregulation, with patients at risk of developing autoimmune diseases or the long‐interval form of MDHS years after the initial episode [26].

In our cohort, four patients developed autoimmune sequelae, including autoimmune thyroiditis, chronic spontaneous urticaria, and membranous glomerulonephritis, over a 10‐year follow‐up, emphasizing the need for prolonged surveillance even after clinical resolution of the acute episode. Notably, these autoimmune complications occurred in patients who had not experienced severe DRESS, and none had pre‐existing autoimmune disease. These findings suggest that autoimmune manifestations may develop independently of the initial clinical severity, highlighting the importance of long‐term monitoring in all patients [26]. This aligns with previous literature suggesting that persistent immune activation may underlie delayed autoimmune manifestations.

Long‐term follow‐up of patients with DRESS is crucial, as subsequent drug hypersensitivity reactions may occur years after the initial event, with reported intervals ranging from 2 to 20 years [11]. In our cohort, four patients developed subsequent DRESS episodes 1–7 years after the first episode, two of whom had experienced severe initial disease, suggesting that the risk of further reactions may persist even after clinical recovery.

All affected patients carried drug‐specific HLA risk alleles corresponding to the implicated drugs; however, no specific allele was uniquely linked to MDHS [11, 47].

Collectively, these observations emphasize that management of DRESS extends beyond acute treatment and requires a long‐term, multidisciplinary approach to address both recurrence risk and delayed immune complications.

### 5.1 Limitations

Firstly, the relatively small number of severe DRESS and MDHS cases limits statistical power and generalizability.

Secondly, patient heterogeneity, including age, comorbidities, and concomitant drugs, complicates interpretation of predictive markers such as HLA alleles and MDHS classification.

Thirdly, diagnostic tests were not uniformly applied, which may have influenced accuracy of culprit drug identification. Fourthly, the absence of population‐level HLA frequency data prevents definitive conclusions about genetic risk. Larger, population‐based studies are warranted to validate these associations and guide personalized management strategies.

## 6 Conclusion

Our findings highlight the complexity of DRESS in the context of multiple drug exposure and genetic susceptibility.

PT and LTT were effective for identifying culprit drugs in patients with DRESS, especially in those exposed to multiple drugs. MDHS was more common in severe cases. In MDHS patients, drug associated HLA risk alleles were frequently identified; however, no single HLA allele was present in MDHS. Careful long‐term follow‐up was critical to detect neo‐sensitization, guide safe reintroduction of essential medications, and monitor for the development of autoimmune sequelae, which may emerge months to years after the acute reaction. These findings underscore the importance of a comprehensive, individualized approach to the diagnosis and management of DRESS.

## Author Contributions

**Derya Ünal:** conceptualization, methodology, validation, formal analysis, supervision, project administration, writing – original draft, writing – review and editing. **Osman Ozan Yeğit:** conceptualization, investigation, data curation, formal analysis, visualization, writing – review and editing. **Semra Demir:** investigation, data curation, validation, writing – review and editing. **Bircan Erden:** conceptualization, validation, formal analysis, data curation, supervision. **Pelin Korkmaz:** investigation, data curation, validation, writing – review and editing. **Fatma Savran Oğuz:** conceptualization, investigation, writing – original draft, writing – review and editing, project administration, resources. **Çiğdem Kekik Çınar:** methodology, software, data curation, investigation, visualization, validation. **Sonay Temurhan:** conceptualization, investigation, formal analysis, data curation. **Deniz Eyice Karabacak:** conceptualization, investigation, software, formal analysis. **Ilkim Deniz Toprak:** investigation, data curation, formal analysis, visualization. **Zeynep Kılınç:** investigation, data curation, validation. **Mehmet Sait Yordam:** writing – original draft, visualization, project administration, supervision. **Nevzat Kahveci:** investigation, resources, validation. **Merve Hörmet İğde:** conceptualization, data curation, visualization, writing – original draft. **Şule Çelik Kamacı:** investigation, resources, data curation. **Ayşe Feyza Aslan:** conceptualization, project administration, resources, supervision, data curation. **Mehmet Emin Sezgin:** investigation, data curation, validation. **Esra Kaya:** conceptualization, validation, software, visualization. **Leyla Bölek:** investigation, data curation, resources. **Raif Coşkun:** conceptualization, investigation, writing – original draft, visualization, funding acquisition, project administration, supervision, data curation. **Müge Olgaç:** conceptualization, investigation, visualization, writing – original draft. **Nida Öztop:** investigation, software, conceptualization, writing – review and editing, data curation. **Aslı Gelincik:** conceptualization, investigation, writing – original draft, writing – review and editing, visualization, validation, methodology, software, formal analysis, project administration, supervision, resources, data curation. All authors reviewed and approved the final version of the manuscript.

## Funding

The authors have nothing to report.

## Conflicts of Interest

The authors declare no conflicts of interest.

## Data Availability

The datasets generated and/or analyzed during the current study are available from the corresponding author on reasonable request.
